# MEDAI-LLM-SUMM: a reporting checklist for medical text summarization studies using large language models

**DOI:** 10.3389/fdgth.2026.1761601

**Published:** 2026-03-02

**Authors:** Anna N. Khoruzhaya, Mariya D. Varyukhina, Rustam A. Erizhokov, Ivan A. Blokhin, Roman V. Reshetnikov, Mariya R. Kodenko, Anastasia P. Pamova, Tikhon A. Burtsev, Kirill M. Arzamasov, Olga V. Omelyanskaya, Anton V. Vladzymyrskyy, Yuriy A. Vasilev

**Affiliations:** Research and Practical Clinical Center for Diagnostics and Telemedicine Technologies of the Moscow Health Care Department, State Budget-Funded Health Care Institution of the City of Moscow, Moscow, Russia

**Keywords:** expert consensus, large language models, medical text summarization, patient safety, reporting guidelines, reproducibility

## Abstract

**Background:**

Medical text summarization using large language models (LLMs) has reached an inflection point in 2024–2025, with adapted models demonstrating capability to match or exceed human expert performance in specific tasks. However, critical gaps persist in safety validation, evaluation frameworks, and clinical deployment readiness. A comprehensive review revealed that only 7% of studies conducted external validation and 3% performed patient safety assessments, with hallucination rates ranging from 1.47% to 61.6%. Existing reporting guidelines, including CONSORT-AI, SPIRIT-AI, TRIPOD-LLM, and DEAL, do not adequately address the specific requirements of medical text summarization tasks.

**Objective:**

to develop MEDAI-LLM-SUMM, the first specialized reporting checklist for research on medical text summarization using LLMs, addressing critical gaps in existing reporting standards.

**Methods:**

A modified iterative consensus approach was employed, comprising three sequential stages: (1) a systematic literature review of 216 publications from PubMed and eLibrary (2023–2025) following PRISMA guidelines and an analysis of existing reporting standards (TRIPOD-LLM, DEAL, CONSORT-AI, SPIRIT-AI, TRIPOD + AI, CLAIM, STARD-AI); (2) development of an initial 44-item, 7-section checklist by a supervisory group; (3) three rounds of face-to-face consensus discussions with a multidisciplinary expert panel of 11 specialists (3 radiologists, 2 clinicians, 3 medical informatics experts, 1 biostatistician, and 2 medical LLM developers). The consensus criterion required unanimous agreement from all panel members.

**Results:**

The final MEDAI-LLM-SUMM checklist comprises 24 items organized into six sections: (A) Clinical validity (4 items addressing clinical task definition, expert involvement, hypothesis formulation, and medical expertise requirements); (B) Model Selection (5 items covering model justification, system requirements, deployment environment, LLM-as-judge approach, and prompt documentation); (C) Data (3 items on datasets, reference summaries with expert consensus, and data stratification); (D) Quality Assessment (8 items including evaluation metrics, clinical metrics, expert evaluation, hallucination detection, LLM-judge assessment, sample size justification, pilot testing, and limitations documentation); (E) Safety (2 items on ethical approval and data anonymization); and (F) Data Availability (2 items on code and dataset accessibility). Comparative analysis with six existing reporting standards demonstrated that MEDAI-LLM-SUMM uniquely addresses hallucination assessment requirements, reference summary creation methodology, LLM-as-judge validation protocols, and detailed pilot testing specifications.

## 1 Introduction

The use of large language models (LLMs) for medical text summarization reached a turning point in 2024–2025. While just two years ago such models produced inconsistent summaries, struggled to extract key information with limited context window (1), their current performance matches that of human experts in specific tasks (2). Van Veen D. et al. showed that LLM-generated summaries of radiology reports and clinical records are comparable (45%) or even superior (36%) to those produced by medical experts (3). However, a more in-depth analysis revealed critical issues regarding testing, evaluation, safety, and readiness for clinical use (4).

Several studies have addressed the limitations and shortcomings of LLM summarization. A recent scoping review by Bednarczyk et al. analyzing 30 studies on medical summarization concluded that the field remains “exploratory and limited in scope,” despite significant commercial investment. While all studies conducted internal validation, only 7% (*n* = 2) performed external validation—evaluation on independent datasets from institutions or populations not involved in model development—and only 3% (*n* = 1) included patient safety risks analysis assessing potential clinical harms from model errors. Furthermore, 57% focused exclusively on narrow tasks (e.g., radiology reports), 50% utilized intensive care unit data, and 87% processed only English-language text. Reported hallucination rate ranged from 1.47% to 61.6% depending on the task complexity and evaluation methodology, with 44% of hallucinations potentially impacting diagnosis or patient management (5). This creates a gap between “laboratory” performance and clinical reality. In certain medical tasks (e.g., assigning ICD codes), GPT-4 barely reached 50% accuracy (6). Certain clinical data entry programs [e.g., Dragon Ambient eXperience (DAX) Copilot], claimed to significantly reduce document processing time, have raised scepticism among outpatient physicians due to a lack of efficacy (7).

This underscores the importance of developing and deploying tools to guide and report studies on the use of LLM for medical data processing. Existing guidelines, including CONSORT-AI (8), SPIRIT-AI (9), and the recently introduced TRIPOD-LLM (10) and DEAL (11), fail to address some critical aspects of medical text summarization. They focus on general application of LLMs and lack subsections related to hallucinations and methodologies for selecting, testing, and validating summarization models. Furthermore, they lack guidance on how to approach the expert consensus methodology, have no established logic behind selecting and ranking evaluation metrics, and do not specify requirements and rules for pilot testing.

Using the literature data and expert panel conclusions, we developed a MEDAI-LLM-SUMM checklist, a tool for filtering out research papers on LLM-based medical text summarization that addresses critical shortcomings of the existing approaches. This checklist focuses on the content details of LLM summarization studies essential for reproducibility, safety assessment, and clinical applicability.

MEDAI-LLM-SUMM is designed for three primary user groups: (1) researchers conducting studies on LLM-based medical text summarization; (2) journal editors and peer reviewers evaluating manuscript submissions; (3) regulatory bodies and healthcare organizations evaluating commercial LLM summarization tools for clinical deployment.

## 2 Materials and methods

### 2.1 Study design

The checklist was developed using a modified iterative consensus approach (12), comprising three sequential stages: a systematic literature review, development of an initial version by a supervisory group, and an iterative consensus process involving a multidisciplinary expert panel.

### 2.2 Rationale for methodology selection

The modified approach to achieving consensus through open discussions was chosen over the classic anonymized Delphi method (13) due to the specific nature of the task. The checklist resulted from live discussions regarding the details of prompt engineering, model architectures, and hallucination metrics, a challenging task that could hardly be achieved through questionnaires alone. Three rounds of documented discussions ensured transparent decision-making comparable to the Delphi method, while offering the advantage of face-to-face interaction and interdisciplinary integration. The checklist development followed the general principles of the EQUATOR Network (14) for reporting guidelines.

### 2.3 Systematic literature review

The systematic review was conducted in accordance with the PRISMA guidelines (15). A search of PubMed and eLibrary databases for 2023–2025 identified 216 relevant studies. For each study, we reviewed the methodology for the following elements: model selection rationale, prompt disclosure, hallucination detection methods, peer review protocols, safety strategies, and pilot testing. We also evaluated the existing reporting standards [TRIPOD-LLM (10), DEAL (11), CONSORT-AI (8), SPIRIT-AI (9), MI-CLAIM (16), STARD-AI (17)] to identify items applicable to summarization tasks and detect potential shortcomings.

### 2.4 Expert panel composition

A supervisory group of two experts (a medical informatics specialist with experience in radiological AI and a radiologist experienced in clinical trials investigating AI products) developed an initial version of the checklist, which included 44 items grouped into seven main sections: study planning and design, data curation, technical specification refinement, testing, data availability, and documentation.

The preliminary version was presented to a multidisciplinary expert panel of 11 specialists: three radiologists, two clinicians, three medical informatics experts, one biostatistician, and two developers of medical LLMs. The experts were required to have at least two years of relevant experience, publications on digital solutions for medicine, and practical experience with medical AI. On average, the experts possessed more than 3 years of experience and had authored five relevant publications. The 2-year threshold was introduced due to the relative novelty of medical LLM domain (widespread adoption since 2023) and the need to engage specialists working with emerging technologies.

The panel included 11 experts, which is consistent with the Delphi recommendations for homogeneous groups. In such studies, the optimal expert panel size is 10–18 participants. When determining the panel size, rather than using traditional power analysis, we sought to achieve a robust consensus and to limit the contribution of each participant to the final distribution of responses. Narrative reviews indicate that most panels in healthcare consist of 8–23 experts (18). Thus, our panel of 11 experts was designed to secure a balance between the statistical robustness and the consensus feasibility.

### 2.5 Consensus process

The consensus process involved three rounds of in-person discussions, each lasting up to two hours, spaced three weeks apart. The meetings were moderated by a member of the supervisory group, who ensured equal representation of opinions using a round-robin technique, where each participant took turns to present their position prior to the open discussion. The checklist items were discussed one-by-one. The experts were allowed to introduce or remove the items, modify wording, or alter the grouping. The arising disagreements were addressed through additional clarifications or rephrasing; otherwise, the decision was postponed until the next meeting and further literature review. The consensus implied a unanimous agreement by all the 11 panel members.

Between the meetings, the supervisors implemented the agreed-upon changes, draw rationales with supporting literature references, and created a document tracking all modifications.

The key disagreements revolved around prompting strategies (resolved by requiring full prompt disclosure, either in the main text or an appendix), pilot testing (shifted from mandatory to recommended but with a clear indication), and hallucination identification criteria (where consensus required both quantitative evaluation of hallucination frequency and a qualitative evaluation of clinical impact).

The final version of the checklist was unanimously approved by all members of the multidisciplinary expert group.

## 3 Results

### 3.1 Literature review findings

A systematic review of 216 publications identified critical gaps in reporting (Vasilev et al., 2025). Quality assessment using PROBAST criteria identified that 98% (211/216) of studies did not reference any reporting standards, while 89% (192/216) demonstrated high risk of bias, primarily due to inadequate documentation of prompt engineering, model versioning, and evaluation methodology. The common lack of technical details regarding model configurations, prompting strategies, and evaluation protocols makes independent verification virtually impossible.

### 3.2 Consensus outcomes

**Figure 1:**
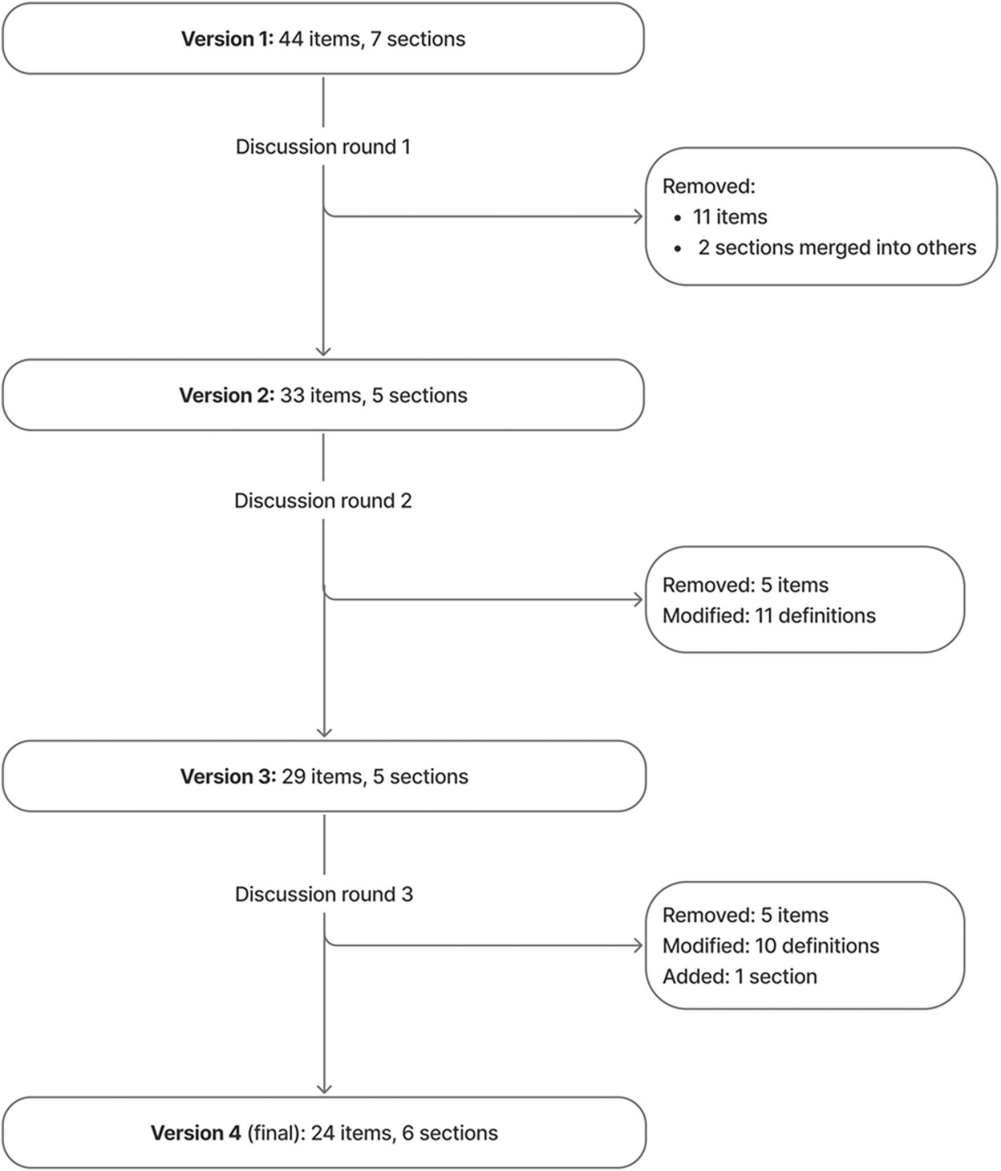
Checklist evolution.

Over the course of three consensus rounds, the checklist evolved from an initial 44 items across 7 sections to a final version comprising 24 items in 6 sections. Key revisions included consolidating overlapping items, removing items deemed unnecessary for context diversity, and adding requirements that emerged during expert discussions (Figure 1).

### 3.3 Final checklist structure

**Figure 2:**
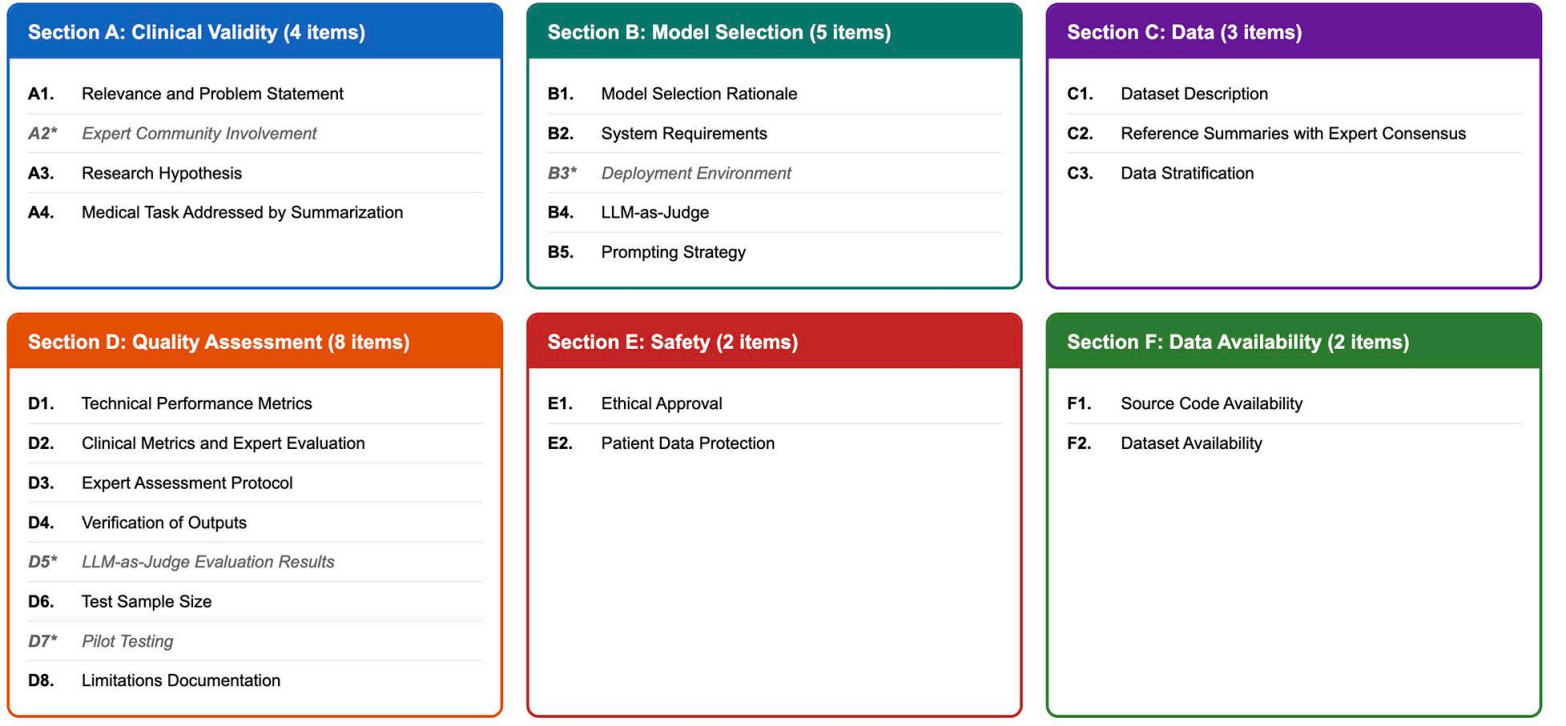
MEDAI-LLM-SUMM checklist structure. Total: 24 items (20 core + 4 optional). Items marked with asterisk are recommended but optional depending on study design.

The MEDAI-LLM-SUMM checklist comprises six sections covering the full lifecycle of medical summarization research, from concept to pilot testing. Each section includes recommended elements that a paper should contain to ensure maximal transparency and reproducibility (Figure 2).

#### 3.3.1 Section A: clinical validity

Medical summarization fundamentally differs from general natural language processing (NLP) tasks due to the need to account for deep clinical context (e.g., extensive chronic disease history), the risks associated with inaccurately summarized information (or the omission of critical details), and the unique healthcare practices across different countries. This section ensures the clinical relevance of the summarization task and the study design prior to technical integration.

A1. General questions to determine the relevance and merit of the study. This section contains three questions related to the clinical task at hand, the availability of similar solutions, and the originality of the proposed method.

A2. Expert community involvement (optional). While this section is optional, the involvement of experts is recommended at all stages: (1) identifying critical domain-specific information that the model must consider; (2) developing evaluation criteria and error classification methodology; (3) output validation through assessing the clinical accuracy and safety of summaries; (4) interpretation using pre-determined error thresholds for specific use cases.

A3. Research hypothesis. The stated hypothesis should clearly specify the object of comparison (e.g., a study scenario comparing LLM summaries with those produced by other machine learning methods or medical experts), the comparison criteria (e.g., evaluation and clinical metrics), and the expected outcome (measurable indicators).

A4. Medical task addressed by summarization. This section is critical as various medical fields share different priorities that render universal quality assessment infeasible. For example, a radiology report summary must preserve the site and size of findings; summaries of medical records must contain vital signs, the onset and course of the patient's condition, and laboratory and imaging data; in oncology, discharge summaries, tumor staging, type of surgical intervention, chemotherapy, and molecular markers are prioritized. Without knowing the clinical context, it is impossible to determine whether a model has missed important information or filtered out redundant details.

#### 3.3.2 Section B: model selection

The quality of a medical text summary depends on the models and their parameters, which indirectly affect the reliability. This section should contain a clear rationale for choosing a specific model, its system requirements, and key configuration parameters critical for the reproducibility.

В1. Rationale for model selection. This section requires disclosure of model details and selection rationale. The rationale should include: a comparative analysis of available models and their suitability for the task; target language support (especially for non-English data); compliance with data privacy requirements and the feasibility of local deployment; outcomes of preliminary or pilot testing (if any) using representative clinical data; and the feasibility of domain-specific fine-tuning through additional training using special medical dictionaries. Where multiple models are considered, the section should contain a rationale for the resulting ensemble.

B2. System requirements. This includes disclosure of GPU/TPU configuration (type, number, VRAM size), system RAM capacity, requirements for data security infrastructure, operating system and driver versions, and data storage requirements for datasets and model versions. This information is critical for study reproducibility at other sites and for the estimation of resource constraints required for scaling.

B3. Deployment environment (optional). For retrained models, this section specifies key hyperparameters (learning rate, batch size, number of epochs, sequence length), the hyperparameter optimization method and search ranges, the optimizer and regularization parameters, seed values for reproducibility, and specific fine-tuning settings (layer freezing strategy, learning rate scheduling, etc.).

B4. LLM-as-a-judge (if applicable). LLM-as-a-Judge refers to the methodology of using one large language model to evaluate the outputs of another. Studies employing this methodology should report: (1) the evaluator model and version, (2) evaluation prompts used, (3) calibration against human judgment with inter-rater reliability metrics, and (4) agreement statistics between LLM and human assessors.

B5. Prompting strategy. Disclosing prompts is critical for the reproducibility of studies that use generative systems. The study should fully disclose the summarization prompts, the prompts used for LLM judge (including detailed assessment criteria and instructions for scale usage), sample prompts with use case examples, and prompt engineering strategies employed to ensure accuracy and safety. All prompts must be available in the main text, the appendix, or in an open repository.

#### 3.3.3 Section C: data

The quality and characteristics of the data govern the reliability and applicability of the study results. This section describes the requirements for the datasets, methodologies for creating reference summaries, and sample stratification.

С1. Datasets. Descriptions of datasets used for domain pre-training and task-specific fine-tuning, the mandatory stages of medical LLM training, must include all document types to be summarized (e.g., medical records, discharge summaries, laboratory and imaging test results, etc.), a statistical rationale for sample size to ensure clinical diversity, and the data type ratio (radiology reports, medical records, etc.).

С2. Reference summaries with expert consensus. This section is expected to detail the reference summary methodology, including: the selection process, the number and credentials of annotators, inter-annotator agreement and disagreement resolution, annotation guidelines, the size and representativeness of the sample of annotated documents, possible alternative approaches (e.g., manual vs. hybrid methods that utilise LLM-generated summaries refined by annotators, or synthetic data), and the rationale for the chosen approach. If existing datasets with reference summaries were used, the section should indicate their source, quality verification, and suitability for the study objectives.

С3. Data stratification. Data stratification may be necessary to achieve balance across different levels of clinical complexity. Unstratified datasets often yield models tailored for typical cases but underperforming in complex clinical scenarios. Disclosing the data stratification contributes to a better understanding of possible reproducibility challenges on similar data that would require specific datasets.

#### 3.3.4 Section D: quality assessment

The quality assessment section should outline a comprehensive methodology for summarizer validation, including evaluation metrics, clinical evaluation by experts, and pilot testing. This section is critical for demonstrating the system's readiness for clinical use and identifying its limitations.

D1. Evaluation metrics relevant to the medical task. The section requires listing the selected metrics (taking into account the task's context) and provide a detailed rationale for their applicability. Traditional NLP metrics, such as ROUGE (which measures n-gram overlap between the generated and reference summary) and BLEU (which assesses word-level accuracy), may be insufficient for the medical context as they focus on superficial similarity rather than preserving clinical meaning. Using an integrated approach requires specifying the methodology for calculating the final metric (e.g., weighted average and minimum value for all components) and provide the results for all the evaluation metrics with 95% confidence intervals (where applicable). The threshold calculation method for the metrics must also be specified.

D2. Clinical metrics and involvement of medical experts. In addition to automated evaluation metrics, expert review of the clinical relevance is critical. The number of experts involved must be statistically justified. The minimum requirement is at least two independent experts for each review. Clinical metrics should reflect the specific domain requirements and may include: preservation of critical information (diagnoses, drug dosages, key tests); absence of clinically significant errors or distortions; time sequence adequately reflecting clinical events; correct medical terminology and compliance with clinical documentation standards; and potential impact on clinical decision-making.

D3. Review by domain experts and validated questionnaire. A validated questionnaire for systematic expert review with clearly defined criteria and scales should be adopted or designed. This section should present a detailed expert review methodology, including: blinding protocols regarding AI-generated and reference summaries; a randomised worklist; statistical methods for inter-rater agreement; and disagreement resolution. The paper should present the review findings indicating the mean values, standard deviations, and confidence intervals for each criterion. In the absence of inter-rater agreement, this decision must be justified or acknowledged as a limitation.

D4. Verification of outputs. An output data verification system is necessary to handle hallucinations. The study is expected to incorporate justified hallucination detection methods (fact-based approaches, entailment-based metrics, LLM-based detection), ensure factual consistency with the original data, verify medical claims against reliable sources (PubMed, clinical guidelines, etc.), and automatically track inconsistencies and unconfirmed claims. Hallucinations class thresholds and security frameworks (guardrails) should be established and outlined.

D5. LLM-as-a-judge evaluation (optional). If the LLM-as-a-judge approach (see Section B4) was used, its parameters and outputs should be disclosed. This should include: the agreement between the LLM and human expert expressed though correlation coefficients and agreement metrics; a detailed overview of the LLM summaries using pre-defined quality criteria; a comparison of LLM robustness using different data subsets; overview of discrepancies between the LLM and experts highlighting possible error patterns; and LLM stability across multiple runs (given the stochastic nature of LLM generation). It is important to note that while LLMs cannot completely replace human expertise, especially when the judgement relies on subtle clinical nuances, they can significantly speed up the evaluation of large volumes of summaries, provided their reliability is adequately validated.

D6. Size of the validation sample. The rationale for the validation sample size should be based on the power analysis that takes into account the expected effect, significance level (usually *α*=0.05), and desired statistical power (usually 80%–90%).

D7. Pilot testing (optional). Evaluation of LLM summarization performance within actual clinical workflows is recommended but recognized as exceeding the scope of typical research projects. Pilot testing should follow a defined design that specifies the study type (laboratory or clinical), duration of follow-up for prospective studies, and key performance indicators (e.g., time-saving for medical record review during diagnosis establishment). Mandatory is the systematic identification and documentation of limitations, including technical constraints (context size, language limitations), clinical limitations (document types, specializations), potential biases, and peer review requirements. These limitations should be clearly articulated to ensure safe implementation in clinical practice.

D8. Limitations are investigated and documented. It should be stated whether the summarizer's limitations have been systematically identified and documented. Clearly defining the limitations is critical for safe implementation in clinical practice and for preventing from using the system in inappropriate contexts. Disclosing the limitations does not detract from the value of the research; on the contrary, it demonstrates a responsible approach and helps practitioners make informed decisions. Limitations should be clearly stated both in the paper and in the user documentation.

#### 3.3.5 Section E: safety

The safety section covers key aspects of ethical and legal compliance during the development, deployment, and further training of AI systems for medical text summarization.

Е1. Ethical approval. Obtaining ethical approval is mandatory for any research using AI to analyze medical data.

E2. Patient Data Protection. Authors must describe the data protection measures implemented, which may include: de-identification/pseudonymization, local model deployment, use of APIs with privacy guarantees, or other technical solutions. Quality control involves continuous monitoring of the LLM's performance using established evaluation systems.

#### 3.3.6 Section F: data availability

This section discusses access to data and model configurations required to assess the reproducibility of the findings. The paper should clearly indicate the availability of the software code and datasets. If each item is present, the access format should be specified (GitHub repository, Zenodo, application, etc.); otherwise, a rationale for the unavailability is required (data confidentiality, license, commercial restrictions, etc.).

### 3.4 Comparison with existing standards

For clarity, we compared the checklist with existing reporting standards (Table 1). The most relevant AI reporting standards in medicine were selected for comparison: TRIPOD-LLM (an LLM-specific tool), DEAL (LLM development and evaluation), MI-CLAIM (clinical applicability of AI), STARD-AI (diagnostic accuracy), CONSORT-AI and SPIRIT-AI (clinical trials with AI).

**Table 1:** Feature comparison across checklists.

Property	MEDAI-LLM-SUMM	Tripod-LLM	Deal	MI-Claim	STARD-AI	Consort-AI	Spirit-AI
Year of introduction	–	2024	2025	2020	2025	2020	2020
Number of key items	24 (6 sections)	19 main items + 50 subitems	2 versions (A and B)	6 main sections	18 new/modified items + STARD 2015	14 additional items + CONSORT 25	15 additional items + SPIRIT 33
Domain	Medical text summarization	Predictive models, wide range of tasks	LLM development and evaluation	Clinical AI models	AI diagnostic accuracy	Clinical trials of AI tools	AI trial protocols
Design methodology	Modified consensus approach, 11 experts	Accelerated Delphi method	Literature review	Expert consensus	Multi-stage process, >240 stakeholders	Consensus	Consensus
Focus on prompt engineering	Detailed (Section B5)	Included	Detailed	Not covered	Not covered	Not covered	Not covered
Hallucination handling	Mandatory (D4)	Mentioned	Mentioned	Not covered	Not covered	Not covered	Not covered
Expert verification	Mandatory with inter-expert agreement	Recommended	Mentioned	Recommended	Recommended	Recommended	Recommended
Pilot testing	Recommended and detailed (D7)	General recommendations	Mentioned	Not covered	Clinical validation	Mandatory	Mandatory
Reference summaries	Detailed requirements (C2)	Not covered	Not covered	Not applicable	Not applicable	Not applicable	Not applicable
LLM-as-a-judge	Detailed requirements (B4, D5)	Mentioned	Mentioned	Not applicable	Not applicable	Not applicable	Not applicable
Clinical validity	Dedicated section A	Integrated	General concepts	Detailed	Detailed	Core element	Core element
Evaluation metrics	Mandatory and summmarization-specific (D1)	Mandatory and task-specific	Mandatory, not detailed	General requirements	Detailed for diagnostics	Trial outcomes	Planned metrics
Interactive tools	None	Available (tripod-llm.vercel.app)	None	None	None	None	None
Scope of application	Summarization only	Prediction, diagnostics, monitoring, screening	All LLM applications	All clinical AI applications	Diagnostics only	All AI interventions in RCTs	All AI trial protocols

## 4 Discussion

The MEDAI-LLM-SUMM checklist addresses a critical gap in medical AI research infrastructure. Our analysis reveals that the 24-item framework captures requirements not adequately covered by existing guidelines, with 8 items (33%) representing entirely novel reporting elements specific to medical text summarization. A recent systematic review of 84 studies found significant heterogeneity in LLM performance across different medical tasks, highlighting the importance of context and task complexity for study planning (19). The proposed MEDAI-LLM-SUMM checklist structure is intended to standardize ongoing medical research and manuscripts based on their findings (the version for work is presented in Supplementary Material 1).

This study focused on the summarization of medical texts. The narrow scope of application was attributed to several factors.

First, summarization is one of the most mature approaches in medical LLM applications, judging by the volume of studies published over the past two years. However, it also raises the most questions regarding the reliability and consistency of approaches to clinical utility (20). A systematic review by Bednarczyk et al. identified serious methodological issues related to widespread neglect of external testing and the virtual absence of patient safety testing (in only 3% of studies) where clinical decisions were intended to rely on AI summaries. For example, 42% of GPT-4 summaries contained hallucinations and 47% missed critical information (5).

In addition, the misleading ease of use (the temptation to reduce the amount of text and leave only the most important information) comes with significant technical complexities that require specific solutions. Tang et al. showed that evaluation metrics correlate weakly with the quality of medical summaries as LLMs are prone to contradictory statements (1). Croxford et al. confirm that in high-risk domains such as healthcare, “good enough” summaries are insufficient due to specific requirements for accuracy and clinical relevance (21). These challenges require specialized evaluation protocols (22) which may rely on customized LLM judges (23).

Third, quality control of summaries requires standardization. While quality and reliability metrics still draw from expert judgement, the ReproNLP study found that the reproducibility of results obtained using experts is understudied, raising concerns for NLP domains where human reference is common, including summarization (24).

All this leads to a peculiar paradox: while the technology is ready for deployment, there are no reliable methods for verifying its safety and effectiveness, despite the apparent need.

MEDAI-LLM-SUMM addresses several critical gaps not covered by existing reporting standards. It provides mandatory requirements for hallucination assessment (item D4), which no other standard explicitly requires. The checklist includes detailed specifications for reference summary creation methodology (item C2), addressing a fundamental challenge in summarization research. It provides specific requirements for LLM-as-judge validation (items B4 and D5), reflecting the growing use of this approach while ensuring rigor. The narrow focus on summarization allows for practical, detailed recommendations rather than general principles characteristic of universal guidelines.

Our approach to this checklist has several methodological limitations that should be considered during interpretation and application.

The most significant limitation is that all 11 members of the expert panel work for multiple organizations in a single country. This creates three types of potential problems: the risk of institutional bias, the limited regulatory context, and the risk of groupthink when reaching consensus. To minimize these risks, the checklist drew from a systematic review of international literature and international standards, rather than institutional preferences. Unanimous agreement by all experts was required, and transparency was ensured by documenting all disagreements and publishing interim versions.

We view the current version as a first iteration requiring international validation. Within 6–12 months of publication, an advisory group with experts from other countries is scheduled to develop version 2.0 based on feedback. The panel size (*n* = 11) is relatively small compared to TRIPOD-LLM or CONSORT-AI, which involved 15–30 experts. However, for the highly specialized task of medical text summarization, the multidisciplinary approach ensured adequate representation of key stakeholders.

Unlike the traditional Delphi method, open discussions were used without anonymizing opinions and without quantitatively grading the scale items (e.g., GRADE 1–9). The use of non-anonymous consensus rounds may have introduced social desirability bias, with participants potentially conforming to opinions of senior colleagues. However, we note that this approach also enabled real-time clarification of complex technical concepts and immediate resolution of terminology disagreements, which was particularly valuable given the novel and rapidly evolving nature of the LLM field. For the version 2.0, we plan to have experts rate the importance of final items on a scale of 1–9 using the GRADE methodology to retrospectively identify critical items.

The most significant methodological limitation of the current version is the lack of formal pilot testing on real publications, which we plan to conduct in the future. Practical applicability, completion time, consistency of item interpretation by different users, comprehensiveness of coverage of real-world methodological issues, and reproducibility of assessments between reviewers were not assessed. The decision to publish the checklist without prior pilot testing was driven by the pressing concern associated with reproducibility in medical LLM research and the adoption of a living document approach, where the current version is considered the first iteration.

We are adopting a regular checklist update approach (similar to TRIPOD-LLM) to maintain relevance amidst the rapid evolution of LLM technologies. Minor updates to clarify wording are planned quarterly as needed, medium updates with item additions are planned annually based on accumulated feedback, and major revisions every 2–3 years or when significant technological changes occur. This iterative approach is consistent with the dynamic nature of the field and allows the checklist to evolve alongside technologies and methodological standards, gradually overcoming the limitations of the initial version.

The MEDAI-LLM-SUMM checklist represents the first specialized reporting standard for medical text summarization studies using LLM. Its implementation will address three fundamental problems: the lack of reproducibility, the absence of a uniform approach to safety assessment, and the gap between laboratory performance and clinical readiness.

The standardized description of studies using the proposed checklist is intended to ensure the reproducibility by detailing the selection of models, prompts, evaluation methodologies, and hallucination detection protocols. The checklist will provide an objective tool for evaluating clinical applicability by incorporating requirements for pilot testing, expert validation, and limitations statement. The standardization of evaluation metrics and testing protocols will provide a methodological framework for regulatory approval and clinical guidelines (a worked example of manuscript evaluation is provided in Supplementary Material 2).

Furthermore, the MEDAI-LLM-SUMM checklist could become a unified methodological tool for study comparison, which is critically important amidst the rapid growth of publications and commercial solutions. The narrow focus on the summarization task allowed us to create detailed and applicable recommendations instead of the general principles typical of universal guidelines. The results can be scaled to related subject areas. The proposed approach can serve as a model for specialized checklists for other medical NLP tasks (diagnostics, treatment planning, clinical coding), gradually covering the entire spectrum of medical LLM applications that share unique features and safety requirements.

MEDAI-LLM-SUMM complements rather than replaces existing guidelines. TRIPOD-LLM provides general reporting standards for clinical prediction models using LLMs, but does not address summarization-specific elements such as reference summary creation methodology, multi-document synthesis, or temporal consistency in longitudinal records. We recommend using MEDAI-LLM-SUMM alongside TRIPOD-LLM for comprehensive reporting.

## Data Availability

The original contributions presented in the study are included in the article/Supplementary Material, further inquiries can be directed to the corresponding author.
