# Supplementary material for: MEDAI-LLM-SUMM: a reporting checklist for medical text summarization studies using large language models
Source: Front Digit Health. 2026 Mar 2;8:1761601. doi: 10.3389/fdgth.2026.1761601 (PMC12989547; doi:10.3389/fdgth.2026.1761601)
Supplement: Supplementary file 1 [file Table1.docx]

Supplementary Material 1

MEDAI-LLM-SUMM: Reporting Checklist for Studies on Medical Text Summarization Using Large Language Models

The MEDAI-LLM-SUMM checklist provides standardized reporting requirements for studies evaluating large language models (LLMs) in medical text summarization tasks. Items marked with an asterisk (*) are recommended but optional depending on study design. For each item, authors should indicate the manuscript section and page number where the information is reported.

# Section A: Clinical validity

| **Item** | **Reporting Requirement** | **Section / Page** |
| --- | --- | --- |
| **A1** | **Relevance and Problem Statement**  Articulate the clinical relevance of the research problem and the scientific novelty of the proposed approach. Provide a systematic review of existing LLM applications addressing similar tasks within the relevant medical domain. |  |
| ***A2**** | ***Expert Community Involvement***  Document stakeholder consultation processes, including structured interviews with domain specialists. Specify the number of participants, their professional qualifications, areas of expertise, and the scope of topics addressed during the planning phase. |  |
| **A3** | **Research Hypothesis**  State the primary research hypothesis with explicit specification of clinical efficacy endpoints and/or technical performance criteria. |  |
| **A4** | **Medical Task Addressed by Summarization**  Define the target medical specialty(ies), enumerate specific clinical tasks, and characterize the source documents subject to summarization (e.g., electronic health records, radiology reports, clinical notes). |  |

# Section B: Model Selection

| **Item** | **Reporting Requirement** | **Section / Page** |
| --- | --- | --- |
| **B1** | **Model Selection Rationale**  Provide comprehensive information on the selected model(s), including justification for selection criteria (e.g., licensing terms, language adaptation, domain-specific pretraining, parameter count, context window limitations). |  |
| **B2** | **System Requirements**  Document the computational infrastructure utilized for model deployment, including hardware specifications (GPU type, memory allocation), software dependencies, and cloud/on-premises configuration. |  |
| ***B3**** | ***Deployment Environment***  Describe the model deployment environment and hyperparameter configuration procedures, including temperature settings, top-p/top-k sampling parameters, and any fine-tuning protocols employed. |  |
| **B4** | **LLM-as-Judge**  State whether an LLM-as-judge methodology was employed (yes/no). If utilized, specify the evaluation tasks, assessment protocol, judge model selection methodology, and provide justification for the chosen approach. |  |
| **B5** | **Prompting Strategy**  Provide complete prompt templates utilized for summarization tasks, including system prompts, user prompts, and any chain-of-thought or few-shot examples. If LLM-as-judge was employed, include all evaluation prompts. |  |

# Section C: Data

| **Item** | **Reporting Requirement** | **Section / Page** |
| --- | --- | --- |
| **C1** | **Dataset Description**  Specify whether publicly available datasets were utilized or proprietary datasets were curated. Provide sample size calculations with appropriate statistical justification, including power analysis where applicable. |  |
| **C2** | **Reference Summaries with Expert Consensus**  Describe the methodology for creating gold-standard reference summaries, including expert consensus procedures, number of annotators, annotation guidelines, and inter-rater reliability assessment. |  |
| **C3** | **Data Stratification**  State whether data stratification was performed (performed / not performed / not applicable). If applicable, describe stratification criteria (e.g., case complexity, document type, clinical specialty, temporal distribution). |  |

# Section D: Quality Assessment

| **Item** | **Reporting Requirement** | **Section / Page** |
| --- | --- | --- |
| **D1** | **Technical Performance Metrics**  Specify automated evaluation metrics selected for the clinical task (e.g., ROUGE, BLEU, BERTScore, clinical-specific metrics) with justification for selection. If composite metrics were employed, describe the aggregation methodology and present disaggregated results. |  |
| **D2** | **Clinical Metrics and Expert Evaluation**  Define clinical outcome measures and describe the involvement of medical domain experts in quality assessment. Provide statistical justification for the number of expert evaluators. |  |
| **D3** | **Expert Assessment Protocol**  State whether a validated assessment instrument was developed (developed / not developed). If developed, describe the expert evaluation protocol, assessment dimensions, rating scales, and inter-rater reliability metrics (e.g., Cohen's kappa, Fleiss' kappa, ICC). |  |
| **D4** | **Verification of Outputs**  State whether output verification procedures for hallucination detection were implemented (yes/no). If implemented, describe the detection methodology, classification taxonomy, and rationale for the selected approach. |  |
| ***D5**** | ***LLM-as-Judge Evaluation Results***  If LLM-as-judge methodology was employed, report evaluation parameters including agreement metrics between automated and human assessment, calibration analysis, and performance outcomes. |  |
| **D6** | **Test Sample Size**  Provide statistical justification for the evaluation sample size, particularly for pilot studies. Include power calculations where applicable. |  |
| ***D7**** | ***Pilot Testing***  State whether pilot testing was conducted (conducted / not conducted). If conducted, describe the study design, testing environment (laboratory / clinical), duration for prospective studies, and preliminary results. |  |
| **D8** | **Limitations Documentation**  State whether usage limitations were systematically identified and documented (yes/no). Include known failure modes, edge cases, and conditions under which model performance may degrade. |  |

# Section E: Safety

| **Item** | **Reporting Requirement** | **Section / Page** |
| --- | --- | --- |
| **E1** | **Ethical Approval**  State whether institutional ethics committee approval was obtained (obtained / not obtained / not applicable). If obtained, provide the protocol reference number and approval date. |  |
| **E2** | **Patient Data Protection**  State whether patient data anonymization or de-identification procedures were implemented (implemented / not implemented). Describe the de-identification methodology and compliance with relevant data protection regulations. |  |

# Section F: Data Availability

| **Item** | **Reporting Requirement** | **Section / Page** |
| --- | --- | --- |
| **F1** | **Source Code Availability**  State whether source code is publicly available (available / not available / available upon request). If available, provide repository URL and licensing information. |  |
| **F2** | **Dataset Availability**  State whether datasets are publicly available (available / not available / available upon request). If available, provide repository URL, data use agreement requirements, and access procedures. |  |

**Notes:**

** Items marked with an asterisk are recommended but optional depending on the study design and objectives.*

*LLM – Large Language Model; ROUGE – Recall-Oriented Understudy for Gisting Evaluation; BLEU – Bilingual Evaluation Understudy; ICC – Intraclass Correlation Coefficient.*
