# Supplementary material for: MEDAI-LLM-SUMM: a reporting checklist for medical text summarization studies using large language models
Source: Front Digit Health. 2026 Mar 2;8:1761601. doi: 10.3389/fdgth.2026.1761601 (PMC12989547; doi:10.3389/fdgth.2026.1761601)
Supplement: Supplementary file 2 [file Table2.docx]

Supplementary Material 2

# Worked example: application of MEDAI-LLM-SUMM Checklist

## Reference Publication

# Van Veen D, Van Uden C, Blankemeier L, et al. Adapted large language models can outperform medical experts in clinical text summarization. Nature Medicine. 2024;30(4):1134-1142. doi:10.1038/s41591-024-02855-5

# Study Overview

This landmark study evaluated eight LLMs across four clinical summarization tasks (radiology reports, patient questions, progress notes, doctor-patient dialogue) using six datasets. A clinical reader study with 10 physicians demonstrated that LLM summaries were preferred to human expert summaries in 81% of cases (equivalent 45%, superior 36%).

**Section A: Clinical Validity**

| **Item** | **Requirement** | **Status** | **Evidence from Publication** |
| --- | --- | --- | --- |
| A1 | Relevance and Problem Statement | ✓ Yes | Clear articulation of clinical burden: physician burnout, documentation time. Systematic review of existing approaches provided. |
| *A2** | *Expert Community Involvement* | ✗ No | No documentation of stakeholder consultation during planning phase. |
| A3 | Research Hypothesis | ✓ Yes | Explicit hypothesis: adapted LLMs can match or exceed medical expert performance in clinical text summarization tasks. |
| A4 | Medical Task Addressed by Summarization | ✓ Yes | Six distinct tasks defined: radiology reports, patient questions, progress notes, doctor-patient dialogue, discharge summaries, and clinical notes. |

**Section B: Model Selection**

| **Item** | **Requirement** | **Status** | **Evidence from Publication** |
| --- | --- | --- | --- |
| B1 | Model Selection Rationale | ✓ Yes | Eight LLMs evaluated with clear selection criteria. GPT-4 identified as primary model with justification based on performance benchmarks. |
| B2 | System Requirements | Partial | API-based deployment mentioned but hardware specifications not documented. Cloud infrastructure implied but not detailed. |
| *B3** | *Deployment Environment* | ✓ Yes | Temperature settings and sampling parameters reported. Domain adaptation procedures described in Methods. |
| B4 | LLM-as-Judge | ✓ Yes | Stated that LLM-as-judge methodology was not employed. Human evaluation used exclusively for quality assessment. |
| B5 | Prompting Strategy | ✓ Yes | Complete prompt templates provided in Supplementary Materials. System prompts and task-specific instructions documented. |

**Section C: Data**

| **Item** | **Requirement** | **Status** | **Evidence from Publication** |
| --- | --- | --- | --- |
| C1 | Dataset Description | ✓ Yes | Six datasets described: MIMIC-III, Open-I, MeQSum, and three proprietary Stanford datasets. Sample sizes provided for each. |
| C2 | Reference Summaries with Expert Consensus | ✓ Yes | Gold-standard references from existing datasets. For clinical reader study, expert physician summaries served as comparators. |
| C3 | Data Stratification | Partial | Stratification by task type and dataset performed. No explicit stratification by case complexity or patient demographics reported. |

**Section D: Quality Assessment**

| **Item** | **Requirement** | **Status** | **Evidence from Publication** |
| --- | --- | --- | --- |
| D1 | Technical Performance Metrics | ✓ Yes | ROUGE-1, ROUGE-2, ROUGE-L, BERTScore reported. Metrics justified for clinical summarization tasks. |
| D2 | Clinical Metrics and Expert Evaluation | ✓ Yes | 10 physicians evaluated summaries across multiple clinical dimensions. Pairwise preference methodology described. |
| D3 | Expert Assessment Protocol | ✓ Yes | Validated assessment instrument with dimensions including completeness, correctness, and clinical utility. Inter-rater reliability reported (κ > 0.8). |
| D4 | Verification of Outputs | Partial | Factual accuracy assessed by physicians but no systematic hallucination detection protocol or taxonomy described. |
| *D5** | *LLM-as-Judge Evaluation Results* | — N/A | Not applicable – LLM-as-judge methodology was not employed in this study. |
| D6 | Test Sample Size | ✓ Yes | Sample sizes justified for each evaluation. Clinical reader study included adequate sample with statistical power considerations. |
| *D7** | *Pilot Testing* | ✗ No | No pilot testing in clinical workflow conducted. Study limited to retrospective evaluation. |
| D8 | Limitations Documentation | ✓ Yes | Limitations section addresses dataset constraints, generalizability concerns, and need for prospective validation. |

**Section E: Safety**

| **Item** | **Requirement** | **Status** | **Evidence from Publication** |
| --- | --- | --- | --- |
| E1 | Ethical Approval | ✓ Yes | Stanford IRB approval documented. Informed consent procedures described for clinical reader study. |
| E2 | Patient Data Protection | ✓ Yes | De-identification procedures described for proprietary datasets. MIMIC-III and Open-I are pre-anonymized public datasets with documented data use agreements. |

**Section F: Data Availability**

| **Item** | **Requirement** | **Status** | **Evidence from Publication** |
| --- | --- | --- | --- |
| F1 | Source Code Availability | ✓ Yes | GitHub repository provided with evaluation scripts and prompt templates. MIT license specified. |
| F2 | Dataset Availability | Partial | Public datasets (MIMIC-III, Open-I, MeQSum) available with standard data use agreements. Proprietary Stanford datasets not available due to privacy restrictions. |

**Summary and interpretation**

*Strengths of reporting*

- Comprehensive model evaluation across eight LLMs with clear selection rationale (B1)
- Rigorous clinical reader study with 10 physician evaluators and validated assessment instrument (D2, D3)
- Complete prompt templates provided in supplementary materials (B5)
- Appropriate ethical oversight and data protection measures documented (E1, E2)
- Source code publicly available enabling reproducibility (F1)

*Gaps identified*

- No systematic hallucination detection protocol or classification taxonomy (D4 – partial)
- Limited documentation of computational infrastructure requirements (B2 – partial)
- No stakeholder consultation documented during planning phase (A2*)
- No pilot testing in clinical workflow environment (D7*)
- Proprietary datasets not available for external validation (F2 – partial)

**Conclusion**

This landmark study demonstrates good overall compliance with MEDAI-LLM-SUMM requirements (71% overall, 80% core items), reflecting its publication in a top-tier journal with rigorous peer review. The primary gaps relate to LLM-specific reporting elements that were not standard practice at the time of publication, particularly systematic hallucination assessment protocols. This worked example illustrates how the MEDAI-LLM-SUMM checklist can identify actionable improvements even in high-quality publications, supporting its utility as both a prospective planning tool and retrospective assessment instrument.

**Compliance summary by section**

| **Section** | **Core Items** | **Optional Items** | **Total** |
| --- | --- | --- | --- |
| A: Clinical Validity | 3/3 (100%) | 0/1 (0%) | 3/4 (75%) |
| B: Model Selection | 3/4 (75%) | 1/1 (100%) | 4/5 (80%) |
| C: Data | 2/3 (67%) | — | 2/3 (67%) |
| D: Quality Assessment | 5/6 (83%) | 0/2 (0%) | 5/8 (63%) |
| E: Safety | 2/2 (100%) | — | 2/2 (100%) |
| F: Data Availability | 1/2 (50%) | — | 1/2 (50%) |
| **TOTAL** | **16/20 (80%)** | **1/4 (25%)** | **17/24 (71%)** |

*Items marked with asterisk (*) are optional. Partial compliance is counted as 0.5 in calculations. D5* rated as N/A (not applicable) as LLM-as-judge was not employed.*
